# Fat compartments in patients with depression: A meta‐analysis

**DOI:** 10.1002/brb3.1912

**Published:** 2020-11-05

**Authors:** Alisa S. Cosan, Julietta U. Schweiger, Kai G. Kahl, Bettina Hamann, Michael Deuschle, Ulrich Schweiger, Anna L. Westermair

**Affiliations:** ^1^ Klinik für Psychiatrie und Psychotherapie Universität zu Lübeck Lubeck Germany; ^2^ LADR Geesthacht Geesthacht Germany; ^3^ Klinik für Psychiatrie, Sozialpsychiatrie und Psychotherapie Medizinische Hochschule Hannover Hannover Germany; ^4^ Kerkhoff Klinik Bad Nauheim Germany; ^5^ Zentralinstitut für Seelische Gesundheit Fakultät für Medizin Mannheim Universität Heidelberg Mannheim Germany

**Keywords:** depression, intra‐abdominal fat, major depressive disorder, metabolic syndrome, subcutaneous fat

## Abstract

**Introduction:**

Depressive disorders are a common illness worldwide. Major depression is known as a significant predictor of the metabolic syndrome. However, the effects of depression on adipose tissue compartments are controversial. This meta‐analysis aimed to evaluate the state of research on the relationship between patients with depression and adipose tissue compartments as compared to nondepressed individuals.

**Methods:**

The PubMed database was searched for human studies that measured adipose tissue compartments such as visceral adipose tissue (VAT), subcutaneous adipose tissue (SAT) and/or organ‐specific adipose tissue measurements using dual‐energy X‐ray absorptiometry, magnetic resonance imaging or computed tomography scan and reported the means and a measure of variance separately for depressed individuals and healthy controls. Twelve articles were identified, including a total of 1,141 depressed and 2,545 nondepressed individuals.

**Results:**

Major depressive disorder and self‐reported depressive symptoms were associated with elevated visceral adipose tissue and elevated subcutaneous adipose tissue. Subanalyses for gender, age, method of adipose tissue measurement, and method of depression assessment showed elevated visceral adipose in depressed individuals. The results could be replicated when focussing on studies controlling for body mass index (BMI). Regarding other adipose tissue compartments, meta‐analysis could not be carried out due to lack of studies.

**Conclusions:**

Depression is associated with enlarged visceral and subcutaneous adipose tissue. Further, especially longitudinal, research is needed to identify the mechanism through which depressive disorders contribute to visceral adiposity.

## Graphical abstract

We conducted a meta‐analysis of studies comparing different adipose tissue compartments between depressed individuals (total *n* = 1,141) and healthy individuals (total *n* = 2,545). Depressed individuals showed elevated visceral adipose tissue, independently of sex, age, method of adipose tissue measurement and method of depression assessment. Subcutaneous adipose tissue was also elevated in depressed compared to healthy individuals.

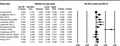

## 1 INTRODUCTION

Depressive disorders are the third leading cause of years lived with disability in both sexes combined (GBD 2017 Disease and Injury Incidence and Prevalence Collaborators, 2018). Depressive symptoms include depressed mood, diminished interest or pleasure, changes in appetite and/or weight, sleep disorders, psychomotor alteration, fatigue, feelings of worthlessness or guilt, concentration problems, and recurrent thoughts of death with or without a specific plan for committing suicide. For a diagnosis of a major depressive disorder five or more of these symptoms have to be present during the same 2‐week‐period and have to cause clinically significant distress (American Psychiatric Association, 2013).

Depressed patients have an increased risk of being or becoming obese, and obese patients have a higher risk of being or becoming depressed—the association between depression and obesity is bidirectional (Mannan et al., 2016). In this regard, abdominal obesity is of special interest because it is the most prevalent manifestation of metabolic syndrome (Despres & Lemieux, 2006). Other manifestations of metabolic syndrome are atherogenic dyslipidaemia, elevated blood pressure, insulin resistance, a proinflammatory state, and a prothrombotic state (Sperling et al., 2015). Abdominal obesity is a marker of “dysfunctional adipose tissue” (Despres & Lemieux, 2006). The accumulation of harmful ectopic fat is associated with cardiovascular diseases—the contribution to the development of coronary artery disease is well established, while emerging evidence pointed out the association with calcific aortic valve disease, atrial fibrillation and left ventricular dysfunction (Mathieu et al., 2014).

As with obesity, major depression is a predictor of the onset and prevalence of metabolic syndrome (Goldbacher et al., 2009). This association is stronger with higher severity of depressive symptoms (Crichton et al., 2016; Hiles et al., 2016). Depressed patients have an increased risk of having or getting a metabolic syndrome, and patients with a metabolic syndrome have an increased risk of being or becoming depressed—the association between depression and metabolic syndrome is bidirectional (Pan et al., 2012).

There is some evidence that patients with severe mental illness display increased amounts of visceral adipose tissue (VAT). Frequently, adipose tissue is divided into the following subcategories: subcutaneous adipose tissue (SAT) and VAT (Kahl et al., 2018). Abdominal obesity frequently describes a summation of SAT and VAT.

Adipose tissue compartments can be assessed using anthropometric scores, such as body mass index, waist‐to‐hip ratio, waist‐to‐height ratio or waist circumference. All of these measures correlate more or less with the amount of total body fat and VAT (Kahl et al., 2018).

There are several techniques for measuring fat compartments, such as dual‐energy X‐ray absorptiometry (DXA), computed tomography (CT) and magnetic resonance imaging (MRI). DXA can be used across the age range, is rapid, and entails relatively low cost and low radiation exposure (Lemos & Gallagher, 2017). However, as DXA only gives a 2D projection, the distribution between VAT and SAT needs to be estimated predicted from an anatomical model (Borga et al., 2018). CT gives a 3D high‐resolution image computed from a large number of X‐ray projections, enabling direct volumetric measurements of adipose tissue depots. In order to minimize subjects’ radiation exposure and because of the labor‐intensity of postprocessing, CT‐based body composition analysis is mostly limited to the 2D analysis of a few axial slices resulting in low precision (Borga et al., 2018). MRI is a nonradiative technique with high sensitivity. However, it is relatively costly, requires participants to remain motionless for the relatively long scan time, and requires specialized postprocessing. MRI is not feasible for persons with claustrophobia (Lemos & Gallagher, 2017).

To sum it up, the bidirectional association of depression and metabolic syndrome may partly be mediated by VAT. Further elucidation of this possible pathway is important given the clinical relevance of both depression and metabolic syndrome. Therefore, we performed a meta‐analysis of studies comparing adipose tissue compartments in patients with vs. without major depressive disorder or self‐reported depressive symptoms.

## 2 METHODS

### 2.1 Sample of studies

**FIGURE 1:**
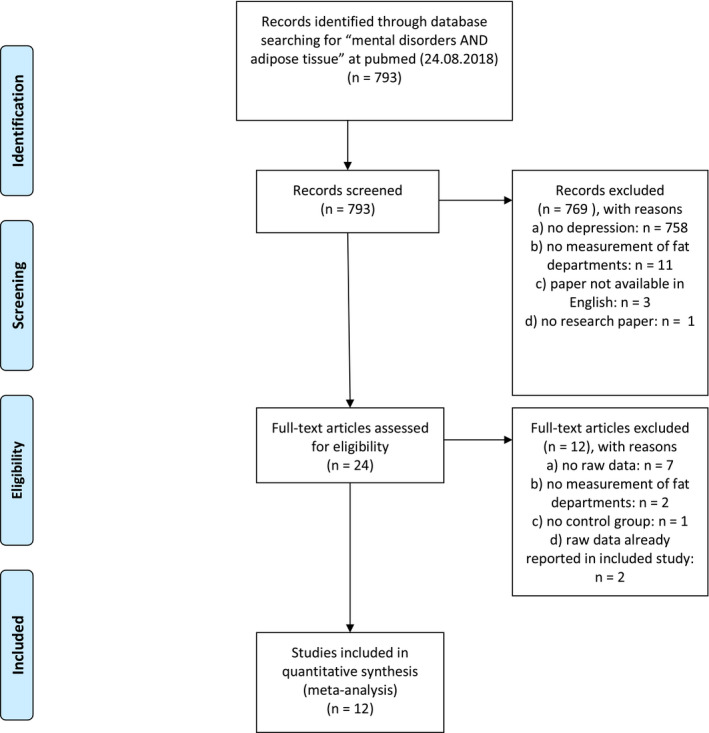
Flow of information according to the PRISMA statement

This meta‐analysis followed the Preferred Reporting Items for Systematic Reviews and Meta‐Analyses (PRISMA) statement, Figure 1 provides the flowchart (Moher et al., 2015). Studies were identified through a comprehensive literature search of the computerized PubMed database from its inception to August 24, 2018 without language restrictions and with the search terms *adipose tissue* and *mental disorders*. The results were restricted to human studies. We first screened each title and abstract of the articles to exclude irrelevant publications and then reviewed the full texts of the remaining articles. The inclusion criteria were as follows: (1) assessment of adipose tissue compartments such as visceral adipose tissue (VAT) and/or subcutaneous adipose tissue (SAT) and/or organ‐specific adipose tissue, (2) comparison of at least one fat compartment between individuals with diagnosed major depressive disorder or self‐reported depressive symptoms and a healthy control group, (3) measurement of fat compartments using dual‐energy X‐ray absorptiometry (DXA), magnetic resonance imaging (MRI) or computed tomography scan (CT), and (4) data on the mean and standard deviation or standard error of the different fat compartments.

### 2.2 Data extraction

Data were extracted using standardized data abstraction forms (see Table 1). The extracted information included the authors’ names, year of publication, country where the study was conducted, sample size, sex and mean age of the participants, depression assessment method, use of antidepressants, fat compartment measured (VAT, SAT, pericardial, paracardial and epicardial adipose tissue), and method of adipose tissue assessment (MRI, CT or DXA).

**TABLE 1:** Characteristics of the studies included in the meta‐analysis

Study	Country	Method	*N*	Sex	mean age ± *SD*	Depression assessment		Fat compartment		Antidepressants
Szczepocka (2018)	Pol	DXA	MDD: 40 HC: 23	f	70.1 ± 4.5 70.1 ± 5.2	ICD−10		VAT, SAT		w
Xiong (2017)	PRC	MRI	MDD: 117 HC: 320	f	58.4 ± 5.0°	SDS ≥ 53		VAT, SAT		n. r.
Coryell (2016)	USA	DXA	MDD: 128 HC: 72	m and f pooled	18.9 ± 1.6 19.1 ± 1.4	DISC‐IV		VAT		w/ wo
Kahl (2014)	Ger	MRI	MDD: 30 HC: 13	m	39.0 ± 10.5 49.1 ± 15.9	SCID for DSM‐IV		VAT, SAT, PeAT, PAT, EAT		w/ wo
			MDD: 20 HC: 12	f	42.2 ± 10.3 44.4 ± 14.3					
Greggersen (2011)	Ger	MRI	MDD: 66 HC: 34	f	34.6 ± 8.2 28.1 ± 6.9	SCID for DSM‐IV		VAT		w/ wo
Ludescher (2011)	Ger	MRI	MDD: 11 HC: 45	f	55.8 ± 7.4°	ICD−10		VAT, SAT		*n*. r.
Vogelzangs (2010)	USA	CT	MDD: 250 HC: 988	m	73.9 ± 2.8 73.6 ± 2.9	CES‐D−10 ≥ 10 or use of antidepressants		VAT		w/ wo
			MDD: 359 HC: 950	f	73.5 ± 2.9 73.5 ± 2.9		
Ludescher (2008)	Ger	MRI	MDD: 10 HC: 12	f	50.3 ± 14.1 50.8 ± 4.4	ICD−10 or DSM‐IV and HAM‐D > 15	VAT		w	
Krishnamurthy (2008)	USA	DXA	MDD: 53 HC: 29	f	36 ± 7 35 ± 7	SCID for DSM‐IV	VAT		w/ wo	
Weber‐Hamann (2006)	Ger	CT	MDD: 29 HC: 17	m and f pooled	61.5 ± 10.5 61.8 ± 9.0	DSM‐IV and HAM‐D ≥ 18	VAT, SAT		wo*	
Weber‐Hamann (2002)	Ger	CT	MDD: 22 HC: 23	f	65.1 ± 8.8 64.0 ± 7.2	DSM‐IV and HAM‐D ≥ 15		VAT		wo*
Thakore (1997)	UK	CT	MDD: 7 HC: 7	f	36.6 ± 4.4 32.7 ± 2.0	DSM‐III‐R and HAM‐D		VAT, SAT		wo


Abbreviations: °, no data reported on subgroups; BDI, Beck's Depression Inventory; BPD, borderline personality disorder; CES‐D, Center for Epidemiologic Studies Depression scale; CT, computed tomography scan; DISC‐IV, Diagnostic Interview Schedule for Children; DSM, Diagnostic and Statistical Manual of Mental Disorders; DXA, dual‐energy X‐ray absorptiometry; EAT, epicardial adipose tissue; f, female; Ger, Germany; HAM‐D, Hamilton Rating Scale for Depression; ICD, International Classification of Diseases; m, male; MDD, major depressive disorder; MRI, magnetic resonance imaging; n.r., not reported; PAT, paracardial adipose tissue; PeAT, pericardial adipose tissue; Pol, Poland; PRC, People's Republic of China; SAT, subcutaneous adipose tissue; SCID, Structured Clinical Interview for the Diagnosis of DSM‐IV disorders; SDS, Zung's Self‐Rating Depression Scale; UK, United Kingdom; USA, United States of America; VAT, visceral adipose tissue; w, with; wo*, without medication a few days before measurement; wo, without.

### 2.3 Statistical analyses

We used Comprehensive Meta‐Analysis (CMA) version 3.0 by Biostat (Borenstein et al., 2013). For each study, the effect size Cohen’s *d* (Cohen, 1988) was calculated according to Lenhard and Lenhard (2016). If a study reported results separate for subgroups such as female and male participants, they were aggregated into a single effect size weighted by subsample size. Thus, each study contributed only one effect size to the main meta‐analysis. A higher weight was given to studies with larger samples; hence, this procedure corrected for the potential bias associated with small sample sizes.

To determine the generalizability of our findings, we quantified the consistency of effects across studies with the *Q* and *I* ^2^ statistics. With regard to *I* ^2^, hetereogeneity can be interpreted as low (25%), moderate (50%) or high (75%) (Higgins et al., 2003). Due to moderate to high heterogeneity within our studies reporting visceral adipose tissue we conducted random effect models for calculation of visceral adipose tissue, whereas studies reporting subcutaneous adipose tissue showed low to moderate heterogeneity so that we conducted fixed effect models for calculation of subcutaneous adipose tissue (Ades et al., 2005; Fleiss, & Gross, 1991).

**FIGURE 2:**
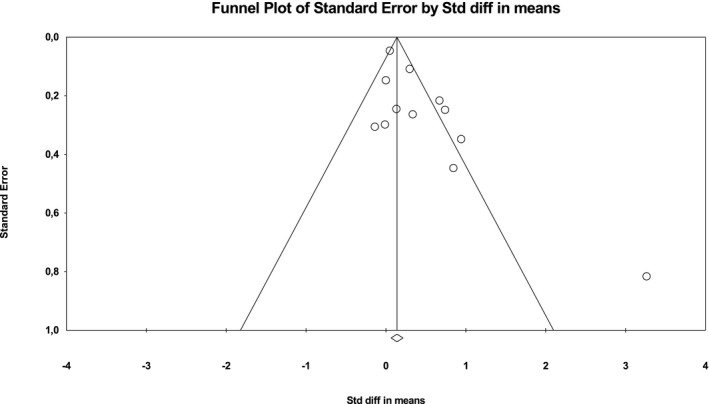
Funnel plot of the studies on visceral adipose tissue in depressed versus non‐depressed subjects

**FIGURE 3:**
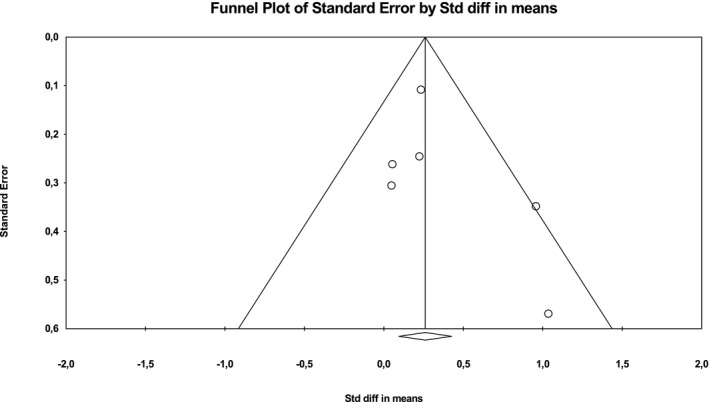
Funnel plot of the studies on subcutaneous adipose tissue in depressed versus non‐depressed subjects

To determine the validity of the meta‐analyses, we employed funnel plots (i.e., plots of the standard difference in means [*d*] against the *SEM*). This was followed by a quantitative evaluation of the degree of asymmetry (Borenstein et al., 2013). The analyses were independently performed for visceral adipose tissue and subcutaneous adipose tissue (see Figures 2 and 3). We then calculated moderator analyses for visceral adipose tissue with method of depression assessment (screening vs. diagnosis), mean age of participants (median‐split of studies according to mean age of participants), measurement method of adipose tissue (CT vs. DXA versus. MRI) and gender (female vs. male) as possible moderators.

To facilitate interpretation of nonsignificant results, we performed post‐hoc power analyses according to Valentine et al. (2010) using the tool “Power calculator for meta‐analysis Version 3” by Jacob Tiebel (https://osf.io/4n6mb/).

## 3 RESULTS

### 3.1 Visceral adipose tissue

**FIGURE 4:**
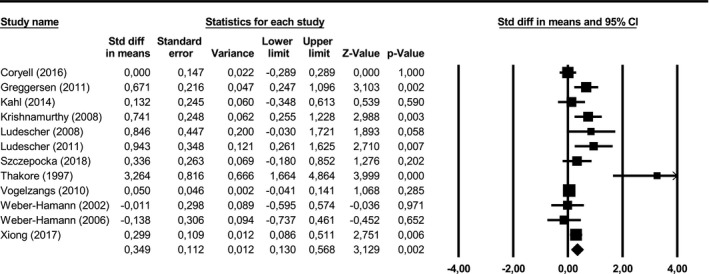
Visceral adipose tissue in depressed versus non‐depressed subjects. CI, confidence interval; Std diff, standardized difference. Positive differences represent greater visceral adipose tissue in depressed subjects compared to non‐depressed subjects

Visceral adipose tissue was significantly larger in depressed subjects than in healthy controls (SMD = 0.35, 95% CI = [0.13; 0.57], *p* = .002) (see Figure 4). Post‐hoc statistical power was 99.95%. In two of the included studies (Krishnamurthy et al., 2008; Xiong et al., 2017), BMI was significantly greater in the depression group than in the control group. As BMI correlates with VAT, we conducted another meta‐analysis without these two studies, which yielded similar results. As there was significant heterogeneity between studies (*Q* = 42.8, *p* < .001, *I* ^2^ = 74.3%), we followed up with moderator analyses.

### 3.2 Moderation by gender

The effect sizes separate for gender were not significantly different (*Q*(1) = 0.8, *p* = .363). In females, visceral adipose tissue in depressed subjects was significantly larger than in healthy controls (SMD = 0.44, 95% CI = [0.17; 0.72], *p* = .002) with significant heterogeneity (*Q* = 41.0, *p* < .001, *I* ^2^ = 78.0%). Post‐hoc statistical power was >99.99%. In males, visceral adipose tissue in depressed subjects was numerically larger but did not significantly differ from healthy controls (SMD = 0.16, 95% CI = [−0.39; 0.71], *p* = .572) without significant heterogeneity (*Q* = 0.002, *p* = .963, *I* ^2^ = 0%). Post‐hoc statistical power was 98.14% (ß = 0.0186) (see Data S1).

### 3.3 Moderation by age

The effect sizes separate for the two age groups were not significantly different (*Q*(1) = 1.8, *p* = .182). In studies on younger participants (pooled mean age 31.5 years, *SD* 7.0 years) visceral adipose tissue was significantly larger in the depressed subjects than in healthy controls (SMD = 0.52, 95% CI = [0.19; 0.86], *p* = .002) with evidence of significant heterogeneity (*Q* = 25.1, *p* < .001, *I* ^2^ = 80.1%). Post‐hoc statistical power was > 99.99%. In studies on older participants (pooled mean age 71.1 years, *SD* 3.7 years) visceral adipose tissue in the depressed subjects did not significantly differ from healthy controls (SMD = 0.22, 95% CI = [−0.09; 0.52], *p* = .160) with significant heterogeneity (*Q* = 11.9, *p* = .036, *I* ^2^ = 58.1%). Post‐hoc statistical power was >99.99% (see Data S2).

### 3.4 Moderation by method of adipose tissue measurement

The effect sizes separate for the three measurement methods were not significantly different (*Q*(2) = 1.0, *p* = .594). Visceral adipose tissue in depressed subjects was numerically larger but did not significantly differ when measuring via CT (SMD = 0.21, 95% CI = [−0.25; 0.67], *p* = .370) and when measuring via DXA (SMD = 0.33, 95% CI = [−0.14; 0.80], *p* = .172) with evidence of significant heterogeneity for CT (*Q* = 15.9, *p* = .001, *I* ^2^ = 81.2%) and for DXA (*Q* = 6.8, *p* = .033, *I* ^2^ = 70.8%). Post‐hoc statistical power for CT measurement was >99.99%, for DXA measurement 65.07%. When measuring via MRT visceral adipose tissue in depressed subjects was significantly larger than in healthy controls (SMD = 0.52, 95% CI = [0.13; 0.90], *p* = .009) without significant heterogeneity (*Q* = 7.1, *p* = .132, *I* ^2^ = 43.4%). Post‐hoc statistical power was >99.99% (see Data S3).

### 3.5 Moderation by method of depression assessment

The effect sizes separate for the two methods for depression assessment were not significantly different (*Q*(1) = 0.9, *p* = .336). When depression was diagnosed by mental health professionals, visceral adipose tissue was significantly larger in depressed subjects than in healthy controls (SMD 0.42, 95% CI = [0.16; 0.69], *p* = .002) with evidence of significant heterogeneity (*Q* = 31.9, *p* < .001, *I* ^2^ = 71.8%). Post‐hoc statistical power was 99.98%. When depression was assessed via screening, visceral adipose tissue in depressed subjects was numerically larger than in healthy controls, but this comparison did not reach significance (SMD = 0.17, 95% CI = [−0.28; 0.62], *p* = .460) with significant heterogeneity (*Q* = 4.4, *p* = .035, *I* ^2^ = 77.5%). Post‐hoc statistical power was 90.61% (see Data S4).

### 3.6 Subcutaneous adipose tissue

**FIGURE 5:**
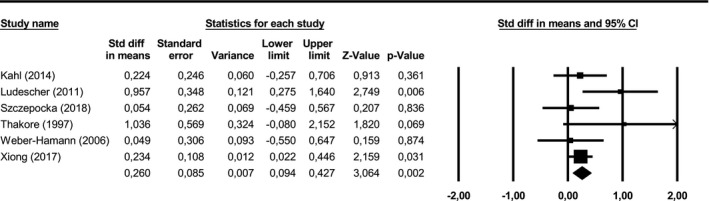
Subcutaneous adipose tissue in depressed versus non‐depressed subjects. CI, confidence interval; Std diff, standardized difference. Positive differences represent greater subcutaneous adipose tissue in depressed subjects compared to non‐depressed subjects

Subcutaneous adipose tissue was significantly larger in depressed subjects than in healthy controls (SMD = 0.26, 95% CI = [0.09; 0.43], *p* = .002) with low to moderate heterogeneity (*Q* = 7.0, *p* = .217, *I* ^2^ = 29.0%) (see Figure 5). Post‐hoc statistical power was 99.97%. In one of the included studies (Xiong et al., 2017), BMI was significantly greater in the depression group than in the control group. As BMI correlates with SAT, we conducted another meta‐analysis without this study, which yielded similar results.

## 4 DISCUSSION

In this meta‐analysis, depression was associated with elevated visceral adipose tissue (VAT) and subcutaneous adipose tissue (SAT). This effect was small to moderate in size for VAT (SMD = 0.35) and for SAT (SMD = 0.26). In moderator analyses, this effect was independent of assessment method of depression, age of participants, measurement method of adipose tissue compartments, and gender.

Examining men and women separately showed increased VAT in both sexes. However, the results were significant with small to medium effect size only for women with major depressive disorders or self‐reported depressive symptoms compared to female healthy controls. While nine studies examined 652 women with major depressive disorder or self‐reported depressive symptoms, only two studies reported data for men with major depressive disorder or self‐reported depressive symptoms (n=280). Further research is necessary to particularly investigate the association between depression in men and visceral adipose tissue.

When the participants were divided by the mean age, the differences between patients with major depressive disorder and self‐reported depressive symptoms and the comparison group were numerically larger in the younger group as well as in the older group. However, only the younger group displayed a significant medium effect size. This may suggest that patients who developed depression earlier in life have a higher risk for increased visceral adipose tissue.

The pooled data showed that visceral adipose tissue between patients with major depressive disorder or self‐reported depressive symptoms and healthy controls was numerically larger in all measurement methods: DXA, MRI and CT. However, only MRI measurement showed a significantly medium effect size. Remarkable, studies with MRI measurement may have less persons with comorbid anxiety spectrum disorders, e.g. claustrophobia, due to measurement conditions. While DXA calculation showed little power, we may failed to see an effect. We found no significant results with CT measurements.

The differences between patients with major depressive disorder or self‐reported depressive symptoms and healthy controls showed significant small to medium effect sizes when the diagnosis was examined by expert interviewers. When depression was assessed via self‐rating scales there was still a small effect between patients with depressive symptom vs. healthy controls but it did not reach for statistical significance. Estimates of depression based on self‐ratings typically yield depression prevalence estimates that are considerably higher than the estimates based on expert interviews. This means that studies based on self‐rating may suffer from a high false positive rate for patients classified as depressed. Consequently, research should be based on reliable diagnoses.

Our findings are of considerable importance as abdominal obesity is the most prevalent manifestation of metabolic syndrome (Despres & Lemieux, 2006), and both metabolic syndrome and depression are highly prevalent in the general population. The American Heart Association recommends assigning depression as a risk factor for adverse medical outcomes in patients with acute coronary syndromes (Lichtman et al., 2014). An altered fat distribution could be a possible link between depression and these effects. One of the studies included in our meta‐analysis reported additional fat compartment measurements, i.e., paracardial, pericardial and epicardial adipose tissue in depressed patients and controls (Kahl et al., 2014, 2017). Pericardial adipose tissue was increased in patients with chronic major depressive disorder compared to controls. Because there was only one database, we could not use these data in our meta‐analysis. For future research, the measurement of these fat compartments as a potential contributing link between depression and cardiac diseases represents a promising approach. None of the included studies recorded any effects of antidepressant medication on VAT. Because drug intake was differently registered and summarized in primary studies we were not able to take antidepressant medication as a moderator in our meta‐analysis.

Due to inclusion criteria and study design two other studies in this field could not be included. However, they are in our opinion worth to mention: Everson‐Rose et al. (2009) published a correlative study with 409 women examined for depressivity and intra‐abdominal adipose tissue using CT. Depressed women showed significantly higher adipose tissue in the intra‐abdominal areas. Lasserre et al. (2014) used subtypes of depression to investigate whether these subtypes are predictive for adiposity and changes in body composition. They found that participants with the atypical subtype of MDD had greater increases in adiposity than participants without MDD; however, VAT was not measured.

When evaluating possible moderators, we must consider that most of the studies were pilot studies. When depression was assessed via diagnosis, the sample sizes in the included studies varied between 7 and 128. These sample sizes have the power to detect only medium‐to‐strong effect sizes and do not allow for multiple regression analysis with a high number of covariates. It remains unclear whether or to what extent behavioral and individual factors, i.e., the use of medication, eating behavior, movement behavior, abuse of substances, somatic and psychiatric comorbidities or duration of illness, contribute to the amount of VAT.

There are several hypotheses about how depressive symptoms might contribute to higher visceral adiposity—a dysregulation of the hypothalamic‐pituitary‐adrenocortical (HPA) system, a developmental origin of health and disease, and adipose tissues as an endocrine organ. First, it is postulated that the development of depression is connected with dysregulation of mineralocorticoid receptors and glucocorticoid receptors. Both of these receptors are part of the HPA system (Holsboer, 2000; Young et al., 2003). For example, patients with major depression with psychosis have higher evening cortisol levels than patients with major depression without psychosis and healthy controls (Keller et al., 2017). A higher severity of hypercortisolism was correlated with higher visceral adiposity (Delivanis et al., 2018). Postulated mechanisms are higher concentrations of glucocorticoid receptors in VAT (Rebuffe‐Scrive et al., 1985), a hypersensitivity of visceral fat adipocytes to cortisol, an increased activity of 11‐beta‐hydroxyreductase activity, and polymorphisms in the glucocorticoid receptor (Ragnarsson et al., 2015). Second, the hypothesis of the developmental origins of health and disease may be relevant. The child of a mother experiencing stress during pregnancy has an increased risk of an altered functioning of the HPA axis (Glover, 2011). Similar mechanisms have been shown in animals (Nyirenda et al., 2009). This could be in line with our result, measuring a significant medium effect size for the younger, but not for the older group. The younger individuals may suffer under alterations of the HPA axis due to the developmental origins of health and disease. Third, adipose tissue is considered one of the largest endocrine organs in the body. Adipose tissue is able to synthesize and release a large number of metabolic products. Excessive visceral fat accumulation causes adipose tissue dysfunctionality. Increased visceral fat is accompanied by adipocyte hypertrophy and hyperplasia, increased inflammation, impaired extracellular matrix remodelling, and fibrosis, together with altered secretion of adipokines (Unamuno et al., 2018). Depressive symptoms are predictive of a higher inflammation status (Hernandez et al., 2018). Furthermore, higher oxidative stress has been demonstrated in patients with depression (Shafiee et al., 2018). There is evidence that genetic variants that increase immune responses are more frequent in patients with depression or characterize a group of individuals at increased risk of developing a depressive phenotype. Concurrently, there is some evidence that increased inflammation is present in a subgroup of depressed patients who were exposed to stress early in childhood or even in utero (Pariante, 2017). Of course, these putative mechanisms are not mutually exclusive and may well work in parallel.

### 4.1 Strengths & limitations

This meta‐analysis was able to identify a substantial number of publications. We found no evidence for publication bias using the funnel plot technique (see Figures 2 and 3). The main limitation was the fact that most of the included studies were pilot studies. A methodological limitation comes from some studies including chronically depressed participants and some studies including participants whose depressive symptoms had lasted only for a short interval prior to measurement. Furthermore, there was a lack of longitudinal data that could enable us to draw further conclusions. Meta‐analysis for other adipose tissue compartments could not be carried out due to lack of studies measuring them. Further research is needed to examine in which way and to what extent depressive symptoms contribute to visceral adiposity.

## 5 CONCLUSION

In this meta‐analysis, depression was associated with enlarged visceral adipose tissue (VAT), independently of sex, age, method of adipose tissue measurement, and method of depression assessment. This could be a hint that elevated VAT may partly mediates the bidirectional association of depression and metabolic syndrome. Also, subcutaneous adipose tissue was enlarged in patients with depression. Further, especially longitudinal, research is needed to identify the mechanism through which depressive disorders contribute to visceral adiposity.

## CONFLICT OF INTERESTS

None.

## AUTHOR CONTRIBUTIONS

JUS, KGK, BH, MD and US designed the study, ASC undertook the literature search and analysis, ASC and AW interpreted the results and drafted the manuscript, and JUS, KGK, BH, MD and US revised the manuscript critically for important intellectual content. All authors approved the final version of the manuscript and agreed to be accountable for all aspects of the work.

## ETHICAL STATEMENT

This work did not involve human or animal subjects.

### Peer Review

The peer review history for this article is available at https://publons.com/publon/10.1002/brb3.1912.

## Supporting information

Data S1

Data S2

Data S3

Data S4

## Data Availability

Data sharing is not applicable to this article as no new data were created or analyzed in this study.
