# Supplementary material for: Fat compartments in patients with depression: A meta‐analysis
Source: Brain Behav. 2020 Nov 5;11(1):e01912. doi: 10.1002/brb3.1912 (PMC7821617; doi:10.1002/brb3.1912)
Supplement: Supplementary file 1 — Data S1 [file BRB3-11-e01912-s001.pdf]

| Group by Gender | Study name | Std diff in means | Standard error | Variance | Lower limit | Upper limit | Z-Value | p-Value |
|---|---|---|---|---|---|---|---|---|
| female | Greggersen (2011) | 0,671 | 0,216 | 0,047 | 0,247 | 1,096 | 3,103 | 0,002 |
| female | Kahl (2014) | -0,235 | 0,366 | 0,134 | -0,953 | 0,483 | -0,641 | 0,522 |
| female | Krishnamurthy (2008) | 0,741 | 0,248 | 0,062 | 0,255 | 1,228 | 2,988 | 0,003 |
| female | Ludescher (2008) | 0,846 | 0,447 | 0,200 | -0,030 | 1,721 | 1,893 | 0,058 |
| female | Ludescher (2011) | 0,943 | 0,348 | 0,121 | 0,261 | 1,625 | 2,710 | 0,007 |
| female | Szczepocka (2018) | 0,336 | 0,263 | 0,069 | -0,180 | 0,852 | 1,276 | 0,202 |
| female | Thakore (1997) | 3,264 | 0,816 | 0,666 | 1,664 | 4,864 | 3,999 | 0,000 |
| female | Vogelzangs (2010) | 0,026 | 0,062 | 0,004 | -0,095 | 0,148 | 0,425 | 0,671 |
| female | Weber-Hamann (2002) | -0,011 | 0,298 | 0,089 | -0,595 | 0,574 | -0,036 | 0,971 |
| female | Xiong (2017) | 0,299 | 0,109 | 0,012 | 0,086 | 0,511 | 2,751 | 0,006 |
| female | | 0,441 | 0,139 | 0,019 | 0,168 | 0,715 | 3,165 | 0,002 |
| male | Kahl (2014). | 0,168 | 0,333 | 0,111 | -0,484 | 0,820 | 0,505 | 0,613 |
| male | Vogelzangs (2010). | 0,152 | 0,071 | 0,005 | 0,013 | 0,291 | 2,149 | 0,032 |
| male | | 0,158 | 0,279 | 0,078 | -0,389 | 0,705 | 0,565 | 0,572 |
| Overall | | 0,385 | 0,125 | 0,016 | 0,140 | 0,629 | 3,084 | 0,002 |

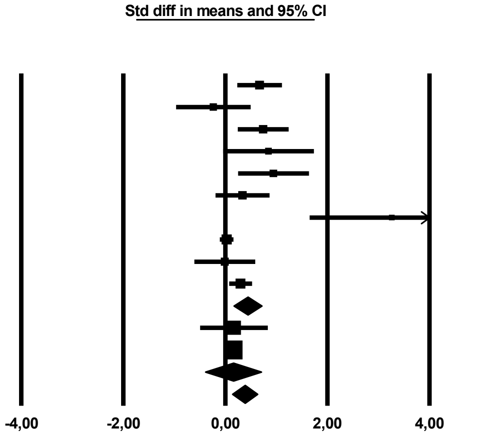


**Supporting Information 1. Visceral adipose tissue in depressed vs. non-depressed subjects, separate for gender.**

Std diff, standardized difference; CI, confidence interval. Positive differences represent enlarged adipose tissue in depressed subjects compared to non-depressed subjects.
