# Supplementary material for: Fat compartments in patients with depression: A meta‐analysis
Source: Brain Behav. 2020 Nov 5;11(1):e01912. doi: 10.1002/brb3.1912 (PMC7821617; doi:10.1002/brb3.1912)
Supplement: Supplementary file 2 — Data S2 [file BRB3-11-e01912-s002.pdf]

Group by Age	Study name	Std diff in means	Standard error	Variance	Lower limit	Upper limit	Z-Value	p-Value
Older	Ludescher (2011)	0,943	0,348	0,121	0,261	1,625	2,710	0,007
Older	Szczepocka (2018)	0,336	0,263	0,069	-0,180	0,852	1,276	0,202
Older	Vogelzangs (2010)	0,050	0,046	0,002	-0,041	0,141	1,068	0,285
Older	Weber-Hamann (2002)	-0,011	0,298	0,089	-0,595	0,574	-0,036	0,971
Older	Weber-Hamann (2006)	-0,138	0,306	0,094	-0,737	0,461	-0,452	0,652
Older	Xiong (2017)	0,299	0,109	0,012	0,086	0,511	2,751	0,006
Older		0,216	0,154	0,024	-0,085	0,518	1,406	0,160
Younger	Coryell (2016)	0,000	0,147	0,022	-0,289	0,289	0,000	1,000
Younger	Greggersen (2011)	0,671	0,216	0,047	0,247	1,096	3,103	0,002
Younger	Kahl (2014)	0,132	0,245	0,060	-0,348	0,613	0,539	0,590
Younger	Krishnamurthy (2008)	0,741	0,248	0,062	0,255	1,228	2,988	0,003
Younger	Ludescher (2008)	0,846	0,447	0,200	-0,030	1,721	1,893	0,058
Younger	Thakore (1997)	3,264	0,816	0,666	1,664	4,864	3,999	0,000
Younger		0,524	0,172	0,029	0,187	0,861	3,051	0,002
Overall		0,361	0,153	0,024	0,060	0,662	2,351	0,019

Statistics for each study

Std diff in means and 95% CI

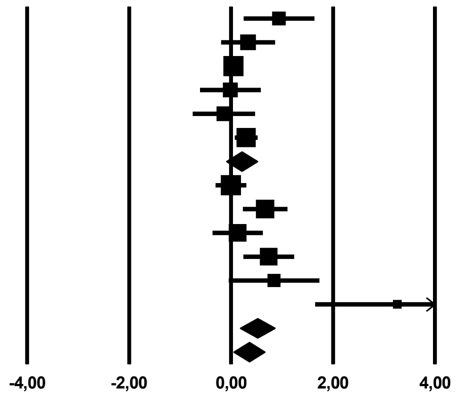

Supporting Information 2. Visceral adipose tissue in depressed vs. non-depressed subjects, separate for age groups.

Std diff, standardized difference; CI, confidence interval. Positive differences represent enlarged adipose tissue in depressed subjects compared to non-depressed subjects.
