# Supplementary material for: Fat compartments in patients with depression: A meta‐analysis
Source: Brain Behav. 2020 Nov 5;11(1):e01912. doi: 10.1002/brb3.1912 (PMC7821617; doi:10.1002/brb3.1912)
Supplement: Supplementary file 3 — Data S3 [file BRB3-11-e01912-s003.pdf]

Group by Measurement	Study name	Std diff in means	Standard error	Variance	Lower limit	Upper limit	Z-Value	p-Value
		Statistics for each study						
CT	Thakore (1997)	3,264	0,816	0,666	1,664	4,864	3,999	0,000
CT	Vogelzangs (2010)	0,050	0,046	0,002	-0,041	0,141	1,068	0,285
CT	Weber-Hamann (2002)	-0,011	0,298	0,089	-0,595	0,574	-0,036	0,971
CT	Weber-Hamann (2006)	-0,138	0,306	0,094	-0,737	0,461	-0,452	0,652
CT		0,209	0,234	0,055	-0,249	0,668	0,896	0,370
DXA	Coryell (2016)	0,000	0,147	0,022	-0,289	0,289	0,000	1,000
DXA	Krishnamurthy (2008)	0,741	0,248	0,062	0,255	1,228	2,988	0,003
DXA	Szczepocka (2018)	0,336	0,263	0,069	-0,180	0,852	1,276	0,202
DXA		0,329	0,241	0,058	-0,143	0,802	1,367	0,172
MRI	Greggersen (2011)	0,671	0,216	0,047	0,247	1,096	3,103	0,002
MRI	Kahl (2014)	0,132	0,245	0,060	-0,348	0,613	0,539	0,590
MRI	Ludescher (2008)	0,846	0,447	0,200	-0,030	1,721	1,893	0,058
MRI	Ludescher (2011)	0,943	0,348	0,121	0,261	1,625	2,710	0,007
MRI	Xiong (2017)	0,299	0,109	0,012	0,086	0,511	2,751	0,006
MRI		0,516	0,198	0,039	0,128	0,904	2,608	0,009
Overall		0,371	0,129	0,017	0,117	0,625	2,867	0,004

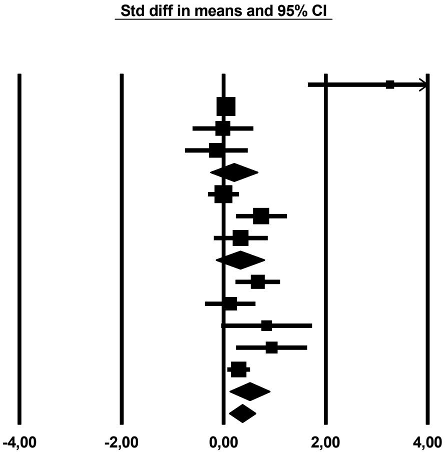

Supporting Information 3. Visceral adipose tissue in depressed vs. non-depressed subjects, separate for methods of adipose tissue measurement.

Visceral adipose tissue measured by dual-energy X-ray absorptiometry (DXA), computed tomography scan (CT) or measured magnetic resonance imaging (MRI) in depressed vs. non-depressed subjects. Std diff, standardized difference; CI, confidence interval. Positive differences represent enlarged adipose tissue in depressed subjects compared to non-depressed subjects.
