# Supplementary material for: Fat compartments in patients with depression: A meta‐analysis
Source: Brain Behav. 2020 Nov 5;11(1):e01912. doi: 10.1002/brb3.1912 (PMC7821617; doi:10.1002/brb3.1912)
Supplement: Supplementary file 4 — Data S4 [file BRB3-11-e01912-s004.pdf]

Group by Assessment	Study name	Statistics for each study							Std diff in means and 95% CI
		Std diff in means	Standard error	Variance	Lower limit	Upper limit	Z-Value	p-Value	
Diagnosis	Coryell (2016)	0,000	0,147	0,022	-0,289	0,289	0,000	1,000	
Diagnosis	Greggersen (2011)	0,671	0,216	0,047	0,247	1,096	3,103	0,002	
Diagnosis	Kahl (2014)	0,132	0,245	0,060	-0,348	0,613	0,539	0,590	
Diagnosis	Krishnamurthy (2008)	0,741	0,248	0,062	0,255	1,228	2,988	0,003	
Diagnosis	Ludescher (2008)	0,846	0,447	0,200	-0,030	1,721	1,893	0,058	
Diagnosis	Ludescher (2011)	0,943	0,348	0,121	0,261	1,625	2,710	0,007	
Diagnosis	Szczepocka (2018)	0,336	0,263	0,069	-0,180	0,852	1,276	0,202	
Diagnosis	Thakore (1997)	3,264	0,816	0,666	1,664	4,864	3,999	0,000	
Diagnosis	Weber-Hamann (2002)	-0,011	0,298	0,089	-0,595	0,574	-0,036	0,971	
Diagnosis	Weber-Hamann (2006)	-0,138	0,306	0,094	-0,737	0,461	-0,452	0,652	
Diagnosis		0,424	0,136	0,019	0,157	0,691	3,113	0,002	
Screening	Vogelzangs (2010)	0,050	0,046	0,002	-0,041	0,141	1,068	0,285	
Screening	Xiong (2017)	0,299	0,109	0,012	0,086	0,511	2,751	0,006	
Screening		0,168	0,228	0,052	-0,279	0,615	0,738	0,460	
Overall		0,357	0,117	0,014	0,128	0,586	3,051	0,002	

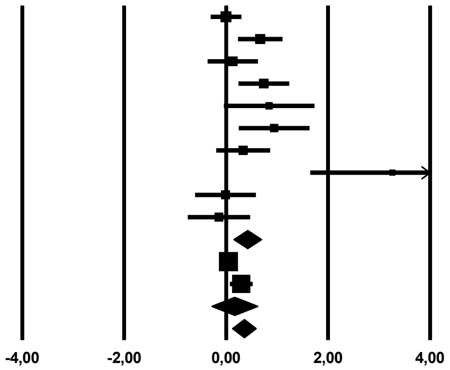

Supporting Information 4. Visceral adipose tissue in depressed vs. non-depressed subjects, separate for methods of depression assessment.

Std diff, standardized difference; CI, confidence interval. Positive differences represent enlarged adipose tissue in depressed subjects compared to non-depressed subjects.
